# Diagnostic Value of Recombinant Heparin-binding Hemagglutinin Adhesin Protein in Spinal Tuberculosis

**DOI:** 10.1515/med-2020-0017

**Published:** 2020-02-28

**Authors:** Feifei Pu, Jing Feng, Fei Niu, Ping Xia

**Affiliations:** 1 Department of Orthopedics, Wuhan No.1 Hospital, Wuhan Integrated TCM & Western Medicine Hospital, Tongji Medical College, Huazhong University of Science and Technology, No.215, Zhongshan Road, Wuhan, Hubei 430022, P.R. China; 2 School of Medicine, Jianghan University, Wuhan, Hubei 430000, P.R. China

**Keywords:** Heparin-binding hemagglutinin adhesin, HBHA, Spinal tuberculosis, Diagnosis

## Abstract

**Background and aim:**

To explore the diagnostic value of recombinant heparin-binding hemagglutinin adhesin (HBHA) protein antigen in spinal tuberculosis.

**Materials and methods:**

Forty patients with spinal tuberculosis were included in the experimental group and 40 healthy people were included in the control group. Serum IgG antibody expression level was detected with recombinant HBHA protein as the antigen, using enzyme-linked immunosorbent assay (ELISA) detection.

**Results:**

Patients with spinal tuberculosis and healthy volunteers were included in this study. A total of 40 eligible patients with spinal tuberculosis were included (24 males and 16 females, aged 18-72 years, with an average age of 41.24 ± 15.74 years). Forty healthy people were included (21 males and 19 females, aged 18-70 years, with an average age of 41.33 ± 12.36 years). On comparing the groups, no significant difference was found in the general data (P >0.05). IgG antibody level in the experimental group was higher than that in the control group, and the difference was significant (P < 0.00001).

**Conclusions:**

Detection of serum HBHA protein antibody is of great value in the auxiliary diagnosis of spinal tuberculosis, and high HBHA expression can be used as an indicator for diagnosis of spinal tuberculosis.

## 1 Introduction

According to the World Health Organization’s global tuberculosis control report of 2011, there were about 8.8 million new tuberculosis cases worldwide in 2010 [1]. Nearly 20 percent of these patients developed extrapulmonary tuberculosis, and 1% to 5% of these patients had spinal tuberculosis [2]. Spinal tuberculosis is a common form of joint tuberculosis and encompasses about 50% of cases of bone tuberculosis, and 1% to 3% of all cases of tuberculosis [3].

Spinal tuberculosis is an occult disease, with the symptoms not being obvious and early diagnosis being difficult. Spinal tuberculosis is more common in young and middle-aged patients, mostly secondary to tuberculosis. The lesions of spinal tuberculosis are mostly located in the lumbar vertebra, followed by the thoracolumbar vertebra, upper thoracic vertebra, cervical vertebra and lumbosacral vertebra. Spinal tuberculosis starts slowly and patients usually seek treatment only when they have severe pain, obvious deformities or neurological symptoms. However, irreversible neurological deficits often occur in the later stages of the disease, so early diagnosis is critical [4]. The detection rates of traditional tuberculosis detection methods (such as tuberculin test, smear staining microscopy, *Mycobacterium tuberculosis* culture, and molecular biology method) for spinal tuberculosis are significantly lower than those for detecting pulmonary tuberculosis [3]. Therefore, it is especially necessary to explore new auxiliary examination methods for spinal tuberculosis.

The heparin-binding hemagglutinin adhesin (HBHA) of *Mycobacterium tuberculosis* is a bacterial protein that is of great significance in the pathogenesis of extrapulmonary tuberculosis through adhesion to epithelial cells [5]. Serum antibody tests have shown a significant value in the diagnosis of tuberculosis [6,7]. In this study, HBHA protein was used to detect HBHA antibody expression levels and explore the diagnostic value of HBHA in spinal tuberculosis.

## 2 Materials and methods

### 2.1 Acquisition of human tissue samples

In the experimental group, specimens were continuously collected between December 2015 and December 2017. During this period, data of 40 patients with spinal tuberculosis were recorded in the Wuhan No.1 Hospital, Wuhan Integrated TCM & Western Medicine Hospital, Tongji Medical College, Huazhong University of Science and Technology. In the patients with spinal tuberculosis, the preoperative imaging examination and serological indexes showed signs of spinal tuberculosis. The preoperative pulmonary imaging showed no obvious signs of tuberculosis infection, and other types of extrapulmonary tuberculosis were excluded. The postoperative histopathological sections of the lesion suggested tuberculosis infection, and the etiological examination showed positive anti-acid staining of the abscess or positive isolation and culture of *Mycobacterium tuberculosis*. In the control group, a series of healthy people were included from December 2015 to December 2017 in the physical examination center of Wuhan No.1 Hospital, Wuhan Integrated TCM & Western Medicine Hospital, Tongji Medical College, Huazhong University of Science and Technology. Tuberculosis, spinal tuberculosis, and other extrapulmonary tuberculosis were excluded by imaging examination.

Patients of the experimental and control groups that had one of the following conditions were excluded from the study: patients with tuberculosis with drug-resistance; patients with diseases affecting immune status, such as infection, trauma, and tumor; patients with tuberculosis of the lungs or other organs that was associated with autoimmune diseases; patients with genetic diseases; individuals who had not received the Bacillus Calmette Guerin vaccine; anyone with a history of tuberculosis contacts.

A total of 40 eligible patients with spinal tuberculosis were included (24 males and 16 females, aged 18-72 years, with an average age of 41.24 ± 15.74 years). Forty healthy people were included (21 males and 19 females, aged 18-70 years, with an average age of 41.33 ± 12.36 years).

### 2.2 Ethical approval and consent to participate

This process was approved by the Ethics Committee of the Wuhan No.1 Hospital, Wuhan Integrated TCM & Western Medicine Hospital, Tongji Medical College, Huazhong University of Science and Technology and conforms to the provisions of the Declaration of Helsinki. Informed consent was obtained from all subjects, and all patients and legal guardians signed the informed consent forms regarding the acquisition of human tissues that were used in this study, in accordance with National Regulations on the Use of Clinical Samples in China. This study met the relevant requirements of the regulations on the administration of medical institutions promulgated by the state council of the People’s Republic of China.

### 2.3 Reagents

Ni Sepharose 6 Fast Flow (ff) His-Tag column and CL-6B column (GE, USA). Protein Maker (Huamei, China). 7h9,7H11 and ADC enrichment broth (Difco, China). HRP-labeled goat anti-mouse IgG (Proteintech, China). The ECL chemiluminescence kit (Biyuntian, China).

### 2.4 Specimen collection and extraction of DNA

Two milliliters of venous blood was extracted using anticoagulant tube extraction in the morning on an empty stomach (Dalian treasure biological engineering co., LTD, China). Leucocyte genome DNA was extracted using this kit. DNA concentration was determined using a UV spectrophotometer and diluted to 100 ng/L in double steaming water.

### 2.5 Preparation of recombinant HBHA protein

E. *coli* BL21 (DE3) competent bacteria were transformed with the recombinant plasmid pETHBHA and were inoculated into Luria Broth (LB) broth (containing kanamycin 50 μg/mL) and cultured at 37°C for 16 hours. The bacteria were expanded at a ratio of 1:20 and cultured at 37°C for two hours (shaking speed 200 r/min). The isopropyl-β-D-thiogalactoside (IPTP) was added to a final concentration of 1 mmol/L, and the culture was maintained at 37°C for 4 hours to induce target protein expression. The induced bacteria were harvested, and the bacterial supernatant was obtained by ultrasonic lysis. The bacterial supernatant was mainly present in a soluble form. The target protein was purified using the non-denaturing Ni Sepharose 6 fast flow (His-Tag) column and subjected to 15% sodium dodecyl sulfate polyacrylamide gel electrophoresis (SDS-PAGE) analysis. The relatively pure protein eluent was collected and dialyzed by gradually reducing the urea concentration in phosphate buffer saline (PBS). The final protein concentration was determined using the bicinchoninic acid (BCA) Protein Concentration Assay Kit and stored in a -80°C freezer.

*M. smegmatis* bacteria were transformed with the recombinant plasmid pMVHBHA, plated on 7H11 agar plates containing kanamycin, and cultured for 5 days. Single clones were picked and inoculated in 7H9 liquid medium containing kanamycin and cultured for 5 days. The bacteria were collected by centrifugation and washed twice in PBS. Bacteria were ultrasonically disrupted. The supernatant was isolated after centrifugation, and the purified protein was obtained by a gradient elution with 0-500 mmol/L sodium chloride in PBS, through a CL-6B column. The final protein concentration was determined using the BCA Protein Concentration Assay Kit, and the protein was aliquoted and stored at -80°C.

### 2.6 HBHA-specific antibody expression level detection

Recombinant HBHA was packaged with an enzyme immunoassay (EIA) enzyme label plate, that was closed after using 3% BSA/PBS. The serum was diluted at a ratio of 1:80, the goat antihuman IgG labeled with horseradish peroxidase was diluted 1:20,000; and then, substrate was added within 30 minutes, using an enzyme standard instrument to measure the optical density (OD) value of specimen. A standard curve was generated to calculate the specimen concentration.

### 2.7 Statistical analysis

In this study, the software SPSS 20.0 (IBM, Chicago, IL, USA) was used to directly compare the OD value of serum antibody titers between different groups. The data presented an approximate normal distribution. *P*< 0.05 was considered statistically significant.

## 3 Results

**Figure 1:**
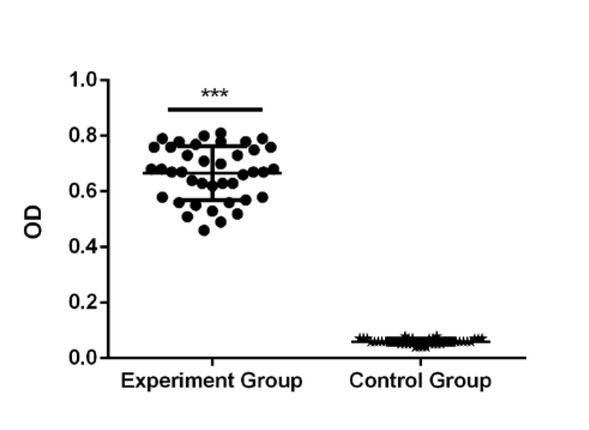
The absorbance of serum HBHA antibody in the spinal tuberculosis group and the healthy control group. The absorbance of antibodies was 0.65 ± 0.16 and 0.06 ± 0.02 in the experimental group and the control group, respectively. A t test was used for statistical analysis, which showed that the difference in HBHA antibody absorbance between the experimental group and the control group was statistically significant (P < 0.00001).

Patients with spinal tuberculosis and healthy volunteers were included in this study, and the comparative analysis showed no significant difference in the general data between the two groups (*P* >0.05) (Table 1). The mean and standard deviation of the serum sample absorbance values in both the groups are listed. In the experimental group, antibody absorbance was 0.65 ± 0.16, whereas in the control group 0.06 ± 0.02. The *t* test was used for statistical analysis, and the HBHA antibody absorbance between the experimental group and the control group was statistically significant (*P* < 0.00001). The absorbance of antibodies is represented by a scatter plot (Figure 1).

**Table 1:** Comparison of general data in two groups

Items	Experimental group	Control group	p-value
Age (year)	41.24±15.74	41.33±12.36	0.98
Male/Female (n/n)	24/16	21/19	0.50
Height (cm)	169.6±6.2	170.4±6.1	0.56
Body mass (kg)	63.6±9.5	63.4±8.1	0.92
Body mass index (kg/cm^2^)	21.8±2.8	21.0±2.4	0.17

## 4 Discussion

Spinal tuberculosis is a type of extrapulmonary tuberculosis and is difficult to diagnose. In fact, the diagnosis of spinal tuberculosis is usually based on clinical manifestations, combined with laboratory tests, imaging examinations, and the exclusion of other causes. It has not been easy to find a positive serum *Mycobacterium tuberculosis* culture. Bacteriological examination is an important method for the diagnosis of spinal tuberculosis, but its culture technology is still lagging behind, the positive rate is low, the current laboratory pathogen detection and identification methods are far from meeting the needs of clinical diagnosis [8]. Lesion biopsy usually has a certain disadvantage and risk factor, and the positive rate is not satisfactory [3,8]. Therefore, the identification of a more reliable diagnostic method for spinal tuberculosis will achieve practical value.

HBHA is a glycoprotein that is expressed and secreted on the surface of *Mycobacterium tuberculosis* and can appear in both bacteria and culture media [9,10]. HBHA binds to receptors containing heparin sulfate on non-phagocytes (such as epithelial cells) through its lysine rich c-terminal; and through this mechanism mediates extrapulmonary tuberculosis [11,12]. Therefore, it is of great significance to explore the value of HBHA in the diagnosis of tuberculosis immunology, especially in the diagnosis of pulmonary tuberculosis.

HBHA is closely associated with the onset of extrapulmonary tuberculosis. The drops degree testing of HBHA-specific IgG antibody in the serum of affected individuals revealed high concentrations of tuberculosis HBHA antibody, while healthy controls typically have low levels (including negative controls and those with latent infections). Increased serum HBHA antibody levels will serve as a marker for diagnosing tuberculosis [9,13].

HBHA has good immunogenicity and can stimulate peripheral blood mononuclear cells with latent infection to produce high levels of IFN-γ. Higher levels of HBHA antibody were detected in the serum of patients with tuberculosis, which suggests that HBHA has higher antigenicity [10,13]. Studies on HBHA have shown that it is associated with the onset of extrapulmonary tuberculosis, but its antibody levels in spinal tuberculosis have not been studied.

This study showed that serum HBHA antibody titers of patients with spinal tuberculosis were higher than those of healthy individuals, and the difference between the two groups was statistically significant. These results suggest that HBHA antibody levels can be used for the auxiliary diagnosis of spinal tuberculosis.

The pathopoiesis of spinal tuberculosis involves the spread of mycobacterium tuberculosis via blood or lymphatic channels; and then, the secretion of bacterial protein results in allergic disease [3]. Tuberculosis antibody expression levels in serum correlate with the growth and metabolic activity of *Mycobacterium tuberculosis* in the body. The body produces a variety of proteins that stimulate the formation of antibodies, and the detection of antibodies can be indirectly reflected by the tuberculosis bacterium growth and metabolic state. These processes can facilitate the diagnosis of tuberculosis [14,15]. However, HBHA protein is related to the incidence of pulmonary tuberculosis, and its corresponding increase in antibody level indicates the release of the HBHA antigen, which indirectly reflects the growth of tuberculosis bacteria [16].

In conclusion, detection of serum HBHA protein antibody is of great value in the auxiliary diagnosis of spinal tuberculosis, and high HBHA expression can be used as an indicator for the diagnosis of spinal tuberculosis.
